# Hybrid Mortar Composites Incorporating Oyster Shell Filler and Recycled Fibers from Disposable Masks

**DOI:** 10.3390/ma18214854

**Published:** 2025-10-23

**Authors:** René Sebastián Mora-Ortiz, Sergio Alberto Díaz Alvarado, Ebelia Del Angel-Meraz, Francisco Magaña-Hernández, Mayra Agustina Pantoja Castro, Emmanuel Munguía-Balvanera

**Affiliations:** División Académica de Ingeniería y Arquitectura (DAIA), Universidad Juárez Autónoma de Tabasco, Carretera Cunduacán-Jalpa de Méndez km. 1, Cunduacán 86690, Tabasco, Mexico; alberto.diaz@ujat.mx (S.A.D.A.); ebelia.delangel@ujat.mx (E.D.A.-M.); francisco.magana@ujat.mx (F.M.-H.); mayra.pantoja@ujat.mx (M.A.P.C.); emmanuel.munguia@ujat.mx (E.M.-B.)

**Keywords:** hybrid materials, eco-friendly mortars, face mask, oyster shell, circular economy

## Abstract

This study presents the development of hybrid masonry mortars by incorporating two waste materials: recycled plastic strips from disposable face masks (FM) as mechanical reinforcement and calcined oyster shell powder (OSP) as a filler. The objective was to evaluate the combined effect of FM and OSP on the mechanical behavior of mortars. Three types of mixes were prepared: a reference mix, a mix with 5% OSP (by cement weight), and mixes with 5% OSP reinforced with FM strips. FM strips were incorporated at three different lengths, dividing the FM-reinforced group into three subgroups (0.1%, 0.2%, 0.5%, and 0.8%). The results showed an approximately 10% increase in compressive strength with the addition of 5% OSP compared to the control mortar, as well as an improvement in bond strength of about 21%. Furthermore, an optimum content of 0.2% of 6 mm strips allowed for adequate dispersion and maintained indirect tensile strengths similar to the control + OSP. OSP acted as a reactive filler, increasing compressive strength and improving both density and adhesion. However, higher FM contents or longer strips increased porosity and water absorption, while reducing strength. This combination represents an innovative strategy for valorizing post-pandemic and marine waste.

## 1. Introduction

The COVID-19 pandemic has led to an unprecedented increase in the use of disposable face masks (FMs), essential for personal protection and virus containment. However, this rise has also generated a significant environmental issue, as most FMs, made from polymers such as polypropylene (PP), are difficult to degrade and may take many years to fully decompose in the environment. This accumulation of plastic waste has resulted in a new source of pollution, mainly affecting aquatic and terrestrial ecosystems [1]. Improperly discarded masks have been found on beaches, rivers, and urban areas, causing severe impacts on wildlife, which may ingest or become entangled in these materials [1,2].

As governments and health institutions promote widespread mask usage, finding solutions for properly disposing of such waste has become an urgent environmental and social challenge [2]. In this context, the reuse of disposable masks as secondary materials in the construction industry has emerged as a promising option to reduce the environmental impact of this waste [3,4]. For instance, Saberian et al. [5] incorporated FM strips cut into 5 × 20 mm as an additive in pavement construction (base/subbase), showing improvements in dry density and uniaxial compressive strength. According to Rajeev et al. [6], the use of PP fibers derived from FMs in 3D-printed concrete leads to higher flexural strength and lower shrinkage. Guo et al. [7], Liu et al. [8], and Selvaranjan et al. [1] point out that due to their hardness, corrosion resistance, and durability, the materials from which FMs are made have the potential to be used in manufacturing masonry bricks, thermal insulation for buildings, moisture barriers, or as elements in concrete mixtures [9,10,11,12]. These researchers also highlight that much research is still needed on all these potential uses for FM waste, as this represents a new area of study.

Several studies have demonstrated that it is possible to decontaminate and reuse surgical and disposable masks through methods such as UV-C radiation, moist/dry heat, or hydrogen peroxide vapor treatment, without significantly affecting the polymer properties [13,14,15,16,17]. These alternatives represent viable strategies to reduce the environmental impact associated with mask use. It should be noted that, for biosafety reasons, some experimental studies have chosen to use new masks to avoid biological risks and ensure fiber integrity [18,19,20]. In this work, new face masks were also used to comply with these restrictions, to ensure sample homogeneity, and to demonstrate the principle of using this waste as reinforcement in mortars.

Within the construction sector, producing masonry mortar stands out as one of the most promising applications for FM recycling. This is because, unlike materials such as concrete, the requirements for mortar in construction codes and standards are significantly less stringent. For example, the minimum compressive strength for structural concrete is set at ≥25–30 MPa (depending on the element type and environmental exposure class). In contrast, for masonry mortars it ranges between ≥2.4 and 17.4 MPa (depending on the type and intended use), in accordance with ASTM C270 [21] and ACI 318 [22]. To date, there have not been many studies on the subject, but researchers such as Ajam et al. [23] concluded that adding FM strips cut into elements of up to 4 cm^2^ in a proportion of up to 5% by volume provides benefits in flexural and compressive strength. Other authors, such as The Win et al. [24] and Miah et al. [25], reported that using FM cut into fibers and added in maximum proportions of 1.5% improves flexural strength and reduces shrinkage. However, compressive strength and water absorption are negatively affected. Nie et al. [26] examined the dynamic properties of mortars incorporating crushed facial masks (5 × 20 mm) up to 0.5 vol%, determining that these additions improved the mechanical performance of the mortar. However, internal defects such as high porosity and low density were observed. The length of the FM fiber added to cement-based mixtures is an important aspect that must be defined. In this context, Idrees et al. [27] used square FM strips of 1.5 to 2.0 mm, observing that in mixtures with these elements, increases in compressive strength were recorded, but decreases in flexural strength occurred. For their part, El Aal et al. [18] reported that recycling 1 cm^2^ FM strips leads to improvements in compressive and flexural strengths. Studies conducted by Avudaiappan et al. [28] suggest that using crushed FM in percentages of up to 1% of the mixture volume increases compressive and split tensile strength by 17% and 22%, respectively. On the other hand, Maloba et al. [20] pointed out that FM strips 5 mm wide and up to 40 mm long added up to 0.5% by volume of mixture improve the flexural strength but affect the compressive strength. Paul et al. [19] reported that using 3 × 42 mm FM strips reduces the concrete strength as the FM content increases but maintains it within acceptable ranges for structural and non-structural applications.

In addition to the growing problem associated with plastic waste, such as FMs, there are organic byproducts whose inadequate management also poses a significant environmental challenge. One such example is oyster shells generated by the aquaculture industry, which, despite their abundance, are often discarded without any valorization process. It is estimated that the shell accounts for approximately 85% of the total weight of an oyster; these shells are frequently stockpiled without proper treatment, causing environmental issues such as visual pollution, foul odors from residual organic matter, and changes in the pH of surrounding soils [29,30,31]. In urban and port areas, poor waste management can quickly turn into a health risk. It allows disease-carrying animals to spread and, over time, lowers the quality of the local environment.

Recycling oyster shells by drying, calcining, and grinding has drawn growing interest. Their composition, rich in calcium carbonate (CaCO_3_), makes them suitable for use in construction materials as a partial substitute for fine aggregates or cement, or simply as an inert mineral filler [32,33,34,35]. Once calcined at temperatures between 700 and 1000 °C, the shells release carbon dioxide and transform into calcium oxide (CaO), which provides a certain binding capacity and a more reactive structure, useful for modifying the mortar microstructure and improving its compactness [36,37,38]. Several studies have shown that the use of oyster shell powder (OSP) in mortar mixes can improve properties such as thermal conductivity, compressive strength, durability against aggressive agents, and reduction in drying shrinkage [33,39,40]. For example, Badreddine et al. [41] reported that incorporating up to 10% calcined oyster shell as filler does not adversely affect mechanical strength and can even reduce the mortar’s capillary absorption. Furthermore, recent research suggests that these fillers can act as nucleating agents in cement hydration, promoting the formation of more homogeneous calcium silicate hydrate (C–S–H) products [42,43]. The utilization of oyster shells not only represents an effective circular economy strategy for the fishing industry but also helps reduce the construction sector’s carbon footprint by partially lowering clinker consumption. Their application as a filler, combined with other waste materials such as plastic fibers from face masks, represents an eco-friendly technological alternative for producing eco-friendly mortars with acceptable performance for non-structural uses. This approach aligns with the principles of eco-design and environmentally oriented materials engineering, integrating criteria of efficiency, waste reuse, and environmental impact reduction into the development of viable construction solutions.

In the pursuit of more eco-friendly construction materials, hybrid mortars have emerged as a promising strategy for integrating multiple waste streams into a single cementitious matrix. These mortars bring together various byproducts: plastic fibers, ashes, marine shells, and even ceramic waste. The goal is to reduce the use of virgin raw materials, enhance mechanical behavior, and lower environmental impacts. The combined use of organic and inorganic waste provides a new way of looking at material recycling in construction, while addressing several ecological problems. This practice is a pillar of eco-design and sustainable materials engineering, as it encourages recycling and contributes to reducing the environmental footprint of waste. In addition to synthetic fibers derived from plastics, several studies have explored the use of natural fibers such as hemp, flax, and sisal in mortars, showing improvements in flexural strength, toughness, and crack control [44,45,46]. Similarly, cellulose fibers derived from industrial waste have been shown to develop a reinforcing effect through microcrack bridging and, when used in moderate contents, improve flexural and post-cracking behavior without compromising compressive strength [47]. However, the performance of these fiber types depends on factors such as their geometry, dosage, surface condition, and the type of matrix used. These factors determine their dispersion and effectiveness as reinforcing mechanisms. For this reason, researchers such as da Costa Santos and Archbold [48] have focused their efforts on developing chemical surface treatments to improve the performance of plant fibers (hemp and flax) in mortar and concrete mixes.

In this context, the combination of disposable mask fibers (FM) with oyster shell powder (OSP) is potentially successful as an eco-friendly option for mortar production. This is because FM and oyster shells are composed of valuable materials, such as polypropylene and calcium carbonate, respectively. Therefore, recycling them not only reduces their accumulation in landfills and natural areas but also extends the life cycle of these materials. The fusion of both in concrete mixes or masonry mortar supports global sustainability goals by driving circular economy practices in the construction sector. Recent studies have focused on analyzing the mechanical and durability behavior of cement-based hybrid mixtures made with additions of various waste materials. This was confirmed by Jahami and Issa [49], who conducted extensive research on the benefits of reusing the six main industrial wastes and their combined use to produce what the authors described as ‘sustainable concrete’. Letelier et al. [50] studied the combined behavior of recycled fine aggregates and glass powder in masonry mortars, showing positive effects on durability and CO_2_ reduction. For their part, Bu et al. [51] presented a review on the use of recycled concrete powder, focusing on cases in which it is combined with other waste such as fly ash or slag to improve the mechanical properties of the mortar. Zhang et al. [52] demonstrated that simultaneously replacing cement and fine aggregates with glass powder and steel slag, respectively, can improve the compressive strength and durability of the mortar. Likewise, Uddin et al. [53] highlighted the potential of hybrid mixtures, demonstrating that using machine learning models, it is possible to accurately predict the compressive strength of what the authors described as sustainable concretes made with combinations of fly ash, slag, silica fume, and other byproducts.

This recycling strategy that promotes the combined use of waste in the production of masonry mortar is aligned with the principles of the United Nations [54], the World Economic Forum [55], and the European Union [56], which promote sustainability and the reduction in the environmental impact of human activities.

This research aims to provide experimental data to contribute to the understanding of the simultaneous effect of adding FM strips as reinforcement and oyster shell powder (OSP) as Filler on the mechanical behavior of masonry mortars. The mortar mixtures were prepared with different percentages of FM (0.1%, 0.2%, 0.5%, and 0.8%), cut into three different sizes (3 × 6 mm, 3 × 15 mm, and 5 × 28 mm), and 5% OSP was added to all of them. For each mixture, changes in compressive and tensile strength, volume of permeable voids, air content, density, water absorption, and shear bond strength were analyzed. The proposed hybrid mortar is intended for non-structural applications in masonry mortars, such as coatings and repair work. This work aims to contribute to reducing the environmental impact of this waste, extending its life cycle, and exploring the benefits that combining both can bring to the performance of construction materials such as mortars and concrete.

## 2. Materials and Methods

**Figure 1:**
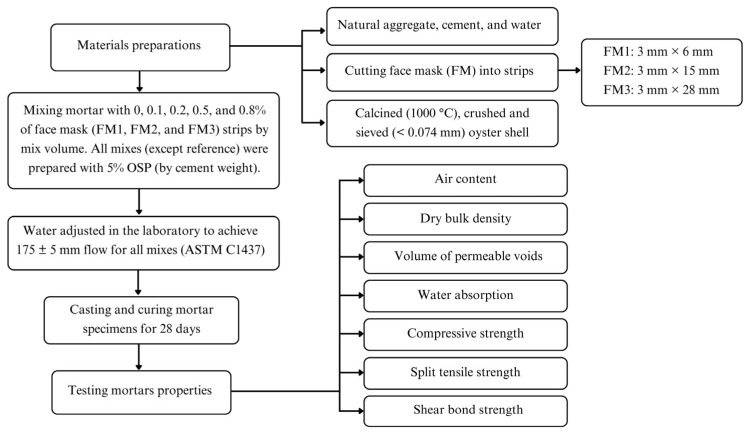
Flowchart of investigation.

Figure 1 shows the flowchart of the investigation.

### 2.1. Disposable Face Mask

**Figure 2:**
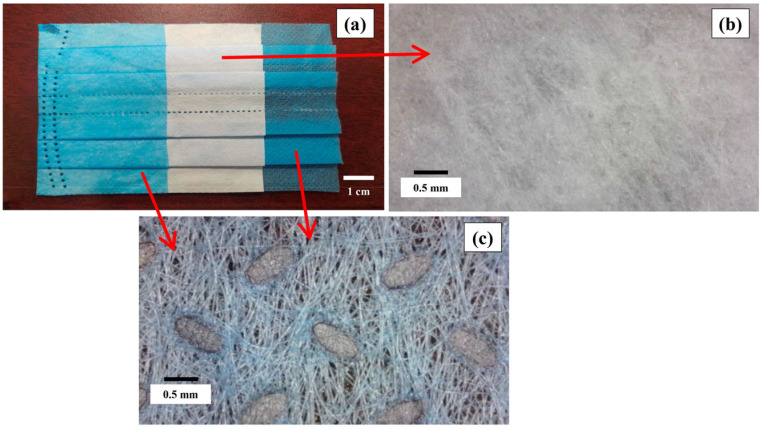
Face mask used: (**a**) three layers of FM; (**b**) nonwoven fabric; and (**c**) melt-blown fabric.

**Figure 3:**
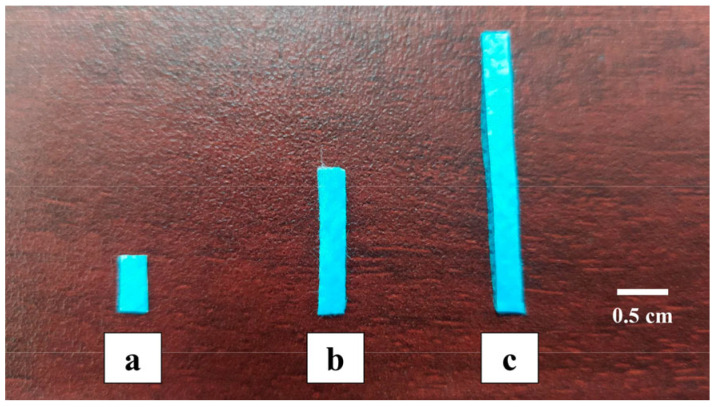
Face mask strips used: (**a**) FM1: 3 × 6 mm; (**b**) FM2: 3 × 15 mm; and (**c**) FM3: 3 × 28 mm.

The face masks used in this research were new and of the same brand. This was performed to avoid sanitary risks [6,12]. Figure 2 shows the three layers that comprise the FM used in this project: the outer layers are nonwoven fabric, while the central layer is melt-blown fabric [57,58]. The manufacturing process provides the layers with high porosity [11,59,60]. The FMs had a thickness of 0.69 ± 0.01 mm, a specific gravity of 0.91 ± 0.01 (ASTM D792 [61]), and water absorption of 6.52 ± 0.09% (ASTM D570 [62]). They were made of isotactic polypropylene (iPP), the most common polymer used in disposable surgical masks, with a weight-average molecular weight (Mw) of approximately 338,000 g/mol, as reported for an iPP mask type by Miller (2025) [63]. FMs were cut into strips of three dimensions: 3 × 6 mm, 3 × 15 mm, and 3 × 28 mm (Figure 3). The strips were labeled FM1, FM2, and FM3, respectively.

### 2.2. Oyster Shell Powder

After being collected from production centers located in the Gulf of Mexico, the shells underwent a process of cleaning, calcination, grinding, and sieving. Cleaning began with full immersion in water for 48 h, followed by manual brushing and natural drying. Calcination was carried out in a Thermo Scientific Lindberg Blue M electric furnace (Thermo Fisher Scientific, Waltham, MA, USA) at 1000 °C for three hours, with a heating and cooling rate of 10 °C per minute. According to studies carried out by Seo et al. [40], calcination of oyster shells at 1000 °C for 3 h allows the complete decomposition of calcium carbonate (CaCO_3_) into calcium oxide (CaO). Studies conducted by Karunadasa et al. [64] and Bui et al. [65] showed that temperatures below 800–850 °C cause a partial conversion of CaCO_3_ to CaO, which reduces the reactivity of the OSP.

After the calcination process, the shells were cooled in a temperature-controlled chamber (25 ± 3 °C), and subsequently, they were ground and sieved until reaching a particle size smaller than 0.075 mm. The grinding process was carried out with a porcelain mortar, while an ASTM No. 200 sieve was used for screening. This entire oyster shell treatment process allows for the reduction in impurities and contaminants in the OSP [66,67].

**Figure 4:**
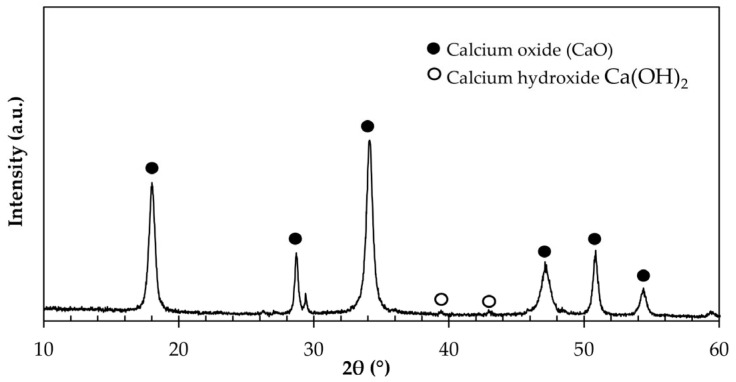
X-ray diffraction (XRD) pattern of OSP used in this research.

As established by ASTM C188 [68], the density of the OSP obtained was 2.53 g/cm^3^. Figure 4 shows the X-ray diffraction (XRD) pattern of the OSP. The diffraction peaks match predominantly with calcium oxide (CaO), accompanied by minor traces of calcium hydroxide Ca(OH)_2_, likely formed by hydration of CaO upon exposure to ambient moisture during handling. These results are consistent with those reported by Seo et al. [40] and Ramón-de los Santos et al. [37], confirming that the selected calcination parameters promote the complete decomposition of CaCO_3_ into CaO without significant formation of secondary crystalline phases. The presence of CaO in the OSP as a dominant mineral is important because, in addition to filling the pores present in the masonry mortar mixtures, it can act as a nucleating agent for cement hydration products. Hence, its effect is double, helping to reduce porosity in the mortar matrix [69].

### 2.3. Cement and Fine Aggregate

**Figure 5:**
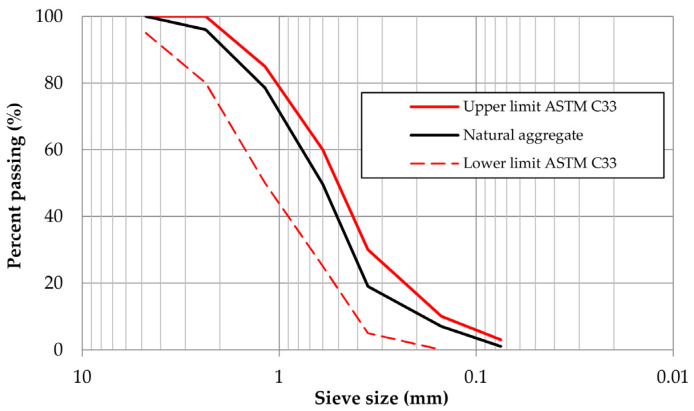
Particle size distribution of natural sand.

River sand was employed as the natural aggregate (NA), with its particle size distribution analyzed following ASTM C33 [70], as shown in Figure 5. Other measured properties included: fine content = 4.6%, specific gravity = 2.66 g/cm^3^, and water absorption = 1.34%. These tests were conducted following ASTM C128 [71] and ASTM C136 [72]. The natural sand used in this study was previously characterized by X-ray diffraction [73], identifying quartz (SiO_2_) and albite (NaAlSi_3_O_8_) as the dominant mineral phases. The median particle size (D_50_), determined from the particle size analysis, was 0.60 mm.

Portland cement type CPO 30R (Type I, high early strength), manufactured by CEMEX, was used in all mortar mixes, complying with the requirements of ASTM C150 [74]. The natural fine aggregate (NA) was a river sand classified according to ASTM C33 [70] as fine aggregate for concrete. Its particle size distribution, presented in Figure 5, yielded a fineness modulus of 3.48.

### 2.4. Mix Proportions

Table 1 shows the dosage of the mortar mixtures used. Five types of samples were designed: base or control sample (Control), control plus 5% OSP as filler (Control + OSP), and one type of mixture for each type of FM strip (FM1, FM2, and FM3). FM strips were incorporated into the mortar mixtures at different percentages by mix volume: 0, 0.1, 0.2, 0.5, and 0.8%. The water content was adjusted in the laboratory to achieve a target flow diameter of 175 ± 5 mm, determined according to ASTM C1437 [75] using the flow table method. In mixtures in which FM was added, the following naming convention was adopted: the letters “FM” indicate that the mixture contains facial mask strip reinforcement; the third digit indicates the type of strip (1, 2, or 3), and at the end of the name, the percentage of the FM reinforcement is indicated. For example, the mixture called FM2-0.5 contains 0.5% of FM2-type strips (3 × 15 mm). Except for the base mix (Control), all other mixes were added with 5% (by dry weight of cement) oyster shell powder (OSP) [40]. The Control + OSP mixture was prepared to evaluate the isolated effect of the filler. This mix had the same proportions as the Control mortar but included 5% OSP by dry weight of cement, without the addition of FM strips.

Previous studies have used low to moderate levels of oyster shell powder (for example, 3–10%) and have shown that at those amounts, strength and other properties are not significantly affected [76,77,78,79]. Based on this information, 5% was chosen as the percentage to use to evaluate the effect of the filler without introducing significant changes in the mortar.

All mixes were prepared as indicated in ASTM C305 [80]: the water and face mask were mixed at 140 rpm for 30 s. Cement was added for 30 s, then the sand was added during another 30 s of mixing. All components were mixed for three minutes, with a 30 s manual mix in between. The fresh mixes were placed in cubic molds (5 cm) and allowed to set in a temperature-controlled room (25 °C ± 3). After 24 h, the specimens were immersed in water for 28 days (curing process).

### 2.5. Testing Program

This section presents the experimental tests carried out to evaluate the mechanical properties of the mortar mixtures. The tests were performed in accordance with ASTM standards.

**Figure 6:**
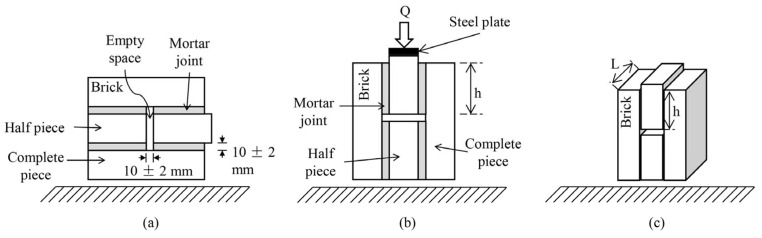
Shear bond strength test setup. Note: (**a**) Construction position; (**b**) testing position; (**c**) dimension reference for shear area calculation.

- Air content: Determined according to ASTM C185 [81] to assess the percentage of entrapped air in fresh mortar.
- Dry bulk density, volume of permeable voids, and water absorption: Determined using 50 mm cubic specimens following ASTM C642 [82].
- Compressive strength: Assessed on 50 mm mortar cubes following ASTM C109 [83].
- Splitting tensile strength: Measured on 100 × 200 mm cylindrical specimens according to ASTM C496 [84]. This test was selected because it allows obtaining a more direct and representative estimate of tensile strength and the effect of fibers on the internal cohesion of the mortar, compared to conventional flexural tests [19,85,86].
- Shear bond strength (SBS): Determined according to standard NMX-C-082-ONNCCE [87]. In determining this property, five specimens of each type of mixture were analyzed. A specimen consists of two brick pieces joined with the mortar mix under analysis (Figure 6). After preparation, the specimens were stored for 28 days in a temperature-controlled laboratory (23 ± 2 °C) and tested using a universal testing machine. The SBS was calculated using Equation (1):

(1) $$SBS=\frac{Q}{A}=\frac{Q}{2Lh}$$

In this equation, *Q* represents the load at failure; A corresponds to the total load-bearing area, while L and h denote the thickness and height of the brick’s mortar-covered surface, respectively.

### 2.6. Statistical Analysis

To validate the influence of recycled materials on the behavior of the mortar, as well as to identify statistically significant differences, an independent samples Student’s *t*-test (Control vs. Control + OSP) and a two-way ANOVA were conducted with a significance level of α = 0.05. When significance existed, Tukey’s Honestly Significant Difference (HSD) post hoc test was performed, which allowed us to establish which mixtures had clear differences between them. For all tests, three specimens per mixture were used (n = 3). In addition to *p*-values, effect sizes were also reported: Cohen’s d for the *t*-test and partial eta squared (η^2^) for the two-way ANOVA. Three statistical analyses were performed with Jamovi version 2.4.8 (The Jamovi Project, Sydney, Australia [88]), an open-source statistical software.

## 3. Results and Discussion

From the analysis of Table 1, which presents the mix proportions under study, it can be observed that adding OSP alone to the Control mixture resulted in a moderate increase in the water-to-cement (W/C) ratio of approximately 3.1% compared to the Control, indicating that this filler slightly increases water demand. When FM strips were incorporated, the W/C ratio showed progressive increases relative to the Control + OSP mixture, as both strip content and length increased, reaching a maximum of nearly 22% above the Control + OSP in the mixture containing 0.8% strips measuring 3 × 28 mm. These variations in W/C are expected to influence the properties of the mortars in both fresh and hardened states, as analyzed in the following sections. The results obtained for the different properties evaluated are presented below, along with their corresponding analysis and discussion.

### 3.1. Air Content

**Figure 7:**
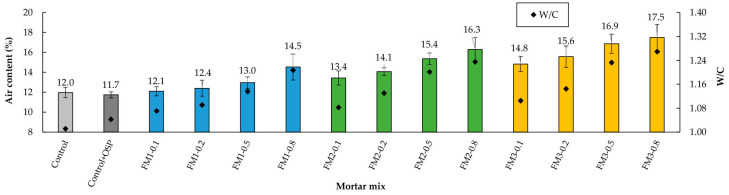
Air content due to the inclusion of FM.

The air content test results showed that, in all three mortar groups, increasing the FM strip content led to higher air content and water-to-cement ratio (W/C) values (Figure 7). This behavior is attributed to the hydrophobic nature of the FM layers, which hinders proper bonding between the cement paste and the strip surface, resulting in air retention at the FM–paste interface. Bhagwat et al. [89] reported that poor bonding between polypropylene (PP) fibers and the concrete matrix generates cracks at the interface, increasing porosity and water absorption. As the number of FM strips increases, their distribution in the mixture becomes less uniform, promoting excessive entanglement between strips, which in turn increases porosity and traps more air bubbles [9,90,91,92]. The increase in W/C occurred because the incorporation of FM strips reduced the workability of the mixtures; to achieve the target consistency for this study (175 ± 5 mm), additional water was required. Similar observations have been reported by other researchers when using PP fibers in concrete samples [93,94,95].

When considering mixtures without FM, the addition of 5% OSP to the Control mortar slightly decreased the air content (≈2.5% relative). This indicates that this filler helps make the mixture pack together more tightly. This reduces the amount of air bubbles that become trapped inside.

Comparing mortars with the same FM content, it was observed that longer FM strips resulted in higher air contents. For instance, when comparing the Control + OSP with mixtures containing 0.1% FM, the air contents for FM1, FM2, and FM3 mortars were approximately 3.4%, 14.5%, and 26.5% higher, respectively. The above indicates that by increasing the length of the strips, the specific surface area and the intertwining between them increase, causing more air bubbles to be trapped in the FM-cement paste interface. The mixtures with the lowest air content were those with the shortest strips and at contents of 0.1% or 0.2% (FM1-0.1 and FM1-0.2).

The *t*-test analysis showed that there are no significant differences in air content between the Control and Control + OSP samples (*p* = 0.536, d = 0.553). This means that adding 5% of filler does not really change how much air becomes trapped in the mixture. The two-way ANOVA analysis showed that fiber content and fiber length both have a significant effect on air content (*p* = 0.001, η^2^ = 0.312; and *p* = 0.001, η^2^ = 0.515, respectively). But the interaction between these two factors was not significant (*p* = 0.933, η^2^ = 0.012). This means their effects work independently of each other. The Tukey test showed that with low content and short lengths, the values are not really different (*p* < 0.05) from Control + OSP. But when content and lengths become higher, there were clear differences compared to Control + OSP.

### 3.2. Dry Bulk Density

**Figure 8:**
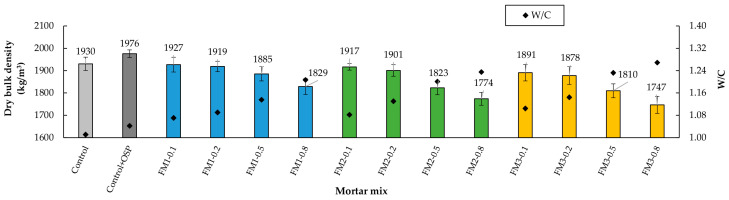
Dry bulk density of the mortars.

As the amount of FM strips increased, a decrease in the dry bulk density of the mortar in all three groups was observed (Figure 8). This reduction is attributed to the low density of the FM strips, the increase in the W/C ratio, and the rise in air content induced by the strips themselves (Section 3.1). These findings align with those observed by other researchers in studies involving PP fibers [24,96].

When analyzing the Control mortar and the Control + OSP mortar, it is observed that the incorporation of the filler slightly increased the apparent density (+2.4% relative); however, the difference was not statistically significant. The above indicates that OSP can improve particle packing and, consequently, reduce pore volume without negatively affecting density. Consequently, although the difference in density was not statistically significant, the inclusion of OSP in the mixtures is justified from a sustainability point of view, since it does not compromise the performance of the mortar.

When comparing mortars with the same FM strip content, dry bulk density decreased as the strip length increased. This suggests that longer fibers contributed to an increase in air content and trip entanglement [93]. For instance, mortars with 0.1% FM content from all three groups (FM1-0.1, FM2-0.1, and FM3-0.1) showed dry bulk density reductions of 2.5%, 2.9%, and 4.3%, respectively, compared to the Control + OSP mortar. Mortars with densities comparable to the control mortar (≤3%) were those with FM strip contents of 0.1% and 0.2% from the FM1 group and 0.1% from the FM2 group.

The *t*-test results showed that the density difference between the Control mortar and the mortar with OSP was not significant (*p* = 0.086, d = −1.849). The ANOVA showed that fiber content and fiber length both matter for dry density (*p* < 0.001, η^2^ = 0.660; and *p* < 0.001, η^2^ = 0.147, respectively). The Tukey test showed that the 0.1% and 0.2% contents were basically the same in all three groups (FM1, FM2, and FM3). But the 0.5% and 0.8% contents were different from each other and also different from the low contents. For length, the mortars with FM1 strips were statistically different from FM2 and FM3. The interaction between content and length was not significant (*p* = 0.693, η^2^ = 0.027).

In summary, the increase in air content, the higher w/c ratio, and the inherent characteristics of the fibers (hydrophobicity and tendency to interlock) largely explain the variations observed later in the hardened state behavior. Therefore, these differences should not be attributed solely to the fiber length.

### 3.3. Volume of Permeable Voids

**Figure 9:**
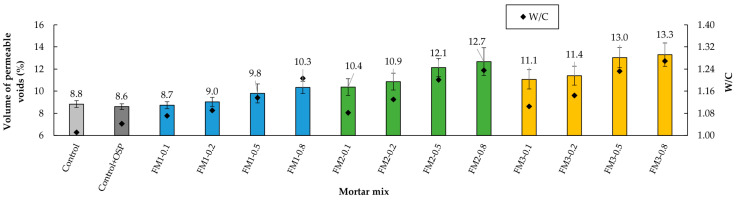
Volume of permeable voids of the mortars.

**Figure 10:**
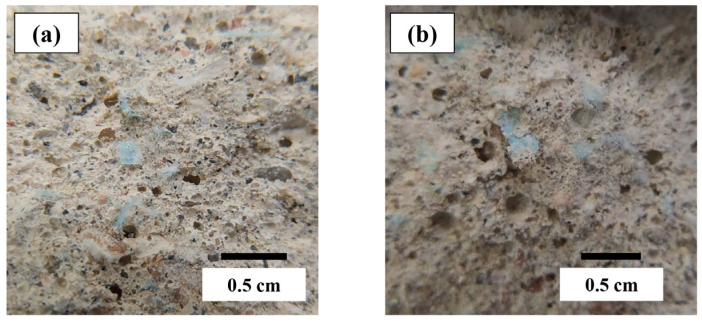
Porosity of samples: (**a**) FM1-0.2 and (**b**) FM1-0.8.

The results of the volume of permeable voids (VPV) test showed that, in the mixtures with FM, as their quantity increased, the pore volume also increased (Figure 9). This trend is closely related to the gradual increase in air content within the mortar mixtures. As mentioned in previous sections, the hydrophobic nature of the FM strips and the entanglements between them generated excess trapped air, leading to a more developed pore structure. Figure 10 compares the porosity developed between the mortars FM1-0.2 and FM1-0.8. These findings are consistent with other studies involving concrete samples reinforced with PP fibers [6,97,98,99]. In these studies, the increase in porosity of concrete samples was associated with a rise in water permeability, which, in turn, was linked to reduced durability of the samples.

When analyzing the Control mortar and the Control + OSP mortar, it is observed that the incorporation of the filler caused a slight reduction in the volume of permeable voids (≈2.3% relative), suggesting that the filler contributed to improving the particle packing.

For mixtures containing FM, the results from the three types of mixtures again showed an adverse effect with increased strip length: longer strips generated higher porosity. As a result, the mortars with the lowest porosity were from the FM1 group, followed by the FM2 group. For example, in mixtures with 0.1% FM content, VPV increased approximately 19.5% from FM1 to FM2 and 27.6% from FM1 to FM3. For an FM content of 0.8% the increase was approximately 23.3% and 29.1%, respectively. Compared to the Control + OSP mortar, the mortars with similar volumes of permeable voids were those containing FM strips of 0.1% and 0.2% from the FM1 type.

The *t*-test analysis showed that there were no significant differences in VPV between the Control mix and the Control + OSP mix (*p* = 0.374, d = 0.882). This proves that this property is not really affected by adding OSP. The ANOVA test results showed that fiber content and strip length had a significant effect on VPV (*p* = 0.001, η^2^ = 0.281; and *p* = 0.001, η^2^ = 0.529, respectively). Just like with air content, the interaction between content and length was not significant (*p* = 0.951, η^2^ = 0.012). The Tukey test showed that low FM contents (0.1% and 0.2%) were statistically similar to each other (*p* ≥ 0.05). But when compared to higher levels like 0.5% and 0.8%, there were clear differences. This means that the effect of fiber content on VPV depends on the amount incorporated. For length, it also showed differences, especially between FM1 versus FM2 and FM3. This supports that both content and length clearly influence pore volume. The statistical tests showed that the interaction effect between length and fiber content is not significant. This means that the influence of length does not depend on the amount of fibers. Each factor acts separately.

### 3.4. Water Absorption

**Figure 11:**
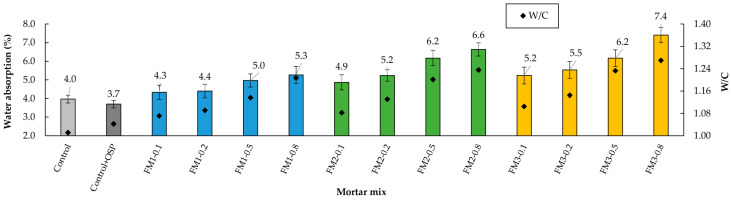
Water absorption of the mortars.

Figure 11 shows the water absorption values for the mortar mixes analyzed. Because water absorption is directly related to the porosity of the samples [89], it is observed in the water absorption results that mortars with higher VPV (Section 3.3) also show high absorption.

Analyzing the behavior of the mixtures without FM, it is observed that the addition of 5% OSP to the Control mortar reduced water absorption by approximately 7.5%. This result corresponds to what was observed in the properties of VPV and dry bulk density, which confirms that OSP can help improve particle packing and reduce the development of the permeable pore network.

During the determination of water absorption in samples with FM, it was noted that as the number and length of FM strips increased, water absorption also rose. Researchers such as Thwe Win et al. [24] concluded that the porosity generated in mortar samples due to the inclusion of PP fibers derived from FM directly affects water absorption. In addition to increased porosity, another factor contributing to the rise in water absorption in mortars with FM strips is the “micro-bridge” effect created by the strips [23,98]. In other words, the length of the strips promotes interconnection between pores, facilitating water migration through different areas of the mortar.

As shown in Figure 11, FM1-type mortars had the lowest water absorption. Within this type, the mortar containing up to 0.2% FM strips exhibited water absorption like the Control + OSP mortar. This suggests that for smaller percentages and lengths of strips, the number of developed pores does not significantly affect water absorption. This allows for better strip distribution and reduces entanglement. Additionally, it helps mitigate the water migration effect caused by micropore bridging. Bhagwat et al. [89] and He et al. [95] reached similar conclusions when studying the impact of adding PP fibers to concrete mixtures.

The *t*-test showed that, regarding water absorption, there was no significant difference between the Control mortar and the mortar with OSP (*p* = 0.185, d = 1.306). The ANOVA analysis showed that fiber content and fiber length had a significant effect on water absorption (*p* < 0.001, η^2^ = 0.460; and *p* < 0.001, η^2^ = 0.367, respectively). The combination of the impact caused by FM content and strip length was not significant (*p* = 0.17, η^2^ = 0.051). The Tukey test showed that the contents of 0.1% and 0.2% were similar (*p* > 0.05) in the three types of mortar (FM1, FM2, and FM3). But when compared with 0.5% and 0.8%, clear differences were found (*p* < 0.05). For length, mortars with FM1 were different from those with FM2 and FM3 (*p* = 0.001). But between FM2 and FM3, there was no difference (*p* = 0.092).

### 3.5. Compressive Strength

**Figure 12:**
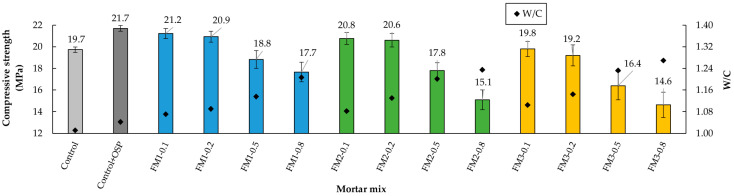
Compressive strength of mortars.

Figure 12 shows that as FM strips were added, the compressive strength decreased in all three types of mortar. This occurred because the increase in FM volume led to a rise in air content, which reduced the density and strength of the samples [24,100]. This tendency was more pronounced in mortar groups with longer strips. This means that longer strips reduced compressive strength [9,101]. For example, compared to the Control + OSP mortar, the compressive strength of mortars with 0.2% FM content dropped by 3.7%, 5.1%, and 11.5% in the FM1, FM2, and FM3 groups, respectively. This behavior occurs because increasing the strip length produces higher porosity in the samples and more pronounced interlocking between the strips. As a result, the development of weak zones at the interface between the mortar paste and the reinforcement strips [11,98,102].

Analyzing the results of mortars without FM, the addition of 5% OSP to the Control mixture increased the compressive strength by 10.2%. This increase indicates that, in addition to the improvement in particle packing and its corresponding increase in the density of the mixtures, adding OSP possibly acted as a nucleating agent for hydration products.

The *t*-test showed that, regarding compressive strength, there was a significant difference between the Control mortar and the mortar with OSP (*p* < 0.001, d = −7.617). This means that adding OSP to the mortar mix improves particle packing and creates a denser matrix. This leads to an increase in compressive strength. The ANOVA analysis showed that fiber content and fiber length had a significant effect on strength (*p* < 0.001, η^2^ = 0.734; and *p* < 0.001, η^2^ = 0.148, respectively). The combination of length and content effects was not significant (*p* = 0.319, η^2^ = 0.028). The Tukey test showed that the contents of 0.1% and 0.2% were similar (*p* > 0.05). But when compared with the contents of 0.5% and 0.8%, clear differences were found (*p* < 0.001). For strip length, mortars with FM1 were different from those with FM2 (*p* = 0.01) and from those with FM3 (*p* < 0.001). This same significant difference was observed between FM2 and FM3 (*p* = 0.014). These results confirm that the negative effect of incorporating FM strips on compressive strength is mainly controlled by their length and content.

**Figure 13:**
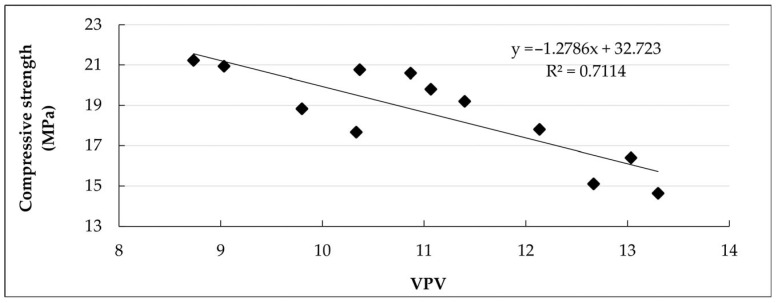
Relationship between volume of permeable voids (VPV) and compressive strength of mortar mixtures.

Figure 13 shows the relationship between VPV and compressive strength for all mortar mixtures analyzed. It is observed that there is a clear inverse relationship (R^2^ = 0.7114): the higher the VPV, the lower the compressive strength. This relationship confirms what was explained in previous sections: increasing the content or length of the strips increases the VPV, which reduces the compressive strength of the mortar. On the other hand, mixtures with lower VPV, such as the Control + OSP mortar, reached higher resistance values.

### 3.6. Split Tensile Strength

**Figure 14:**
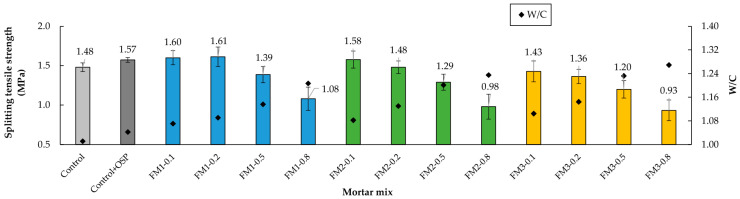
Split tensile strength of mortars.

**Figure 15:**
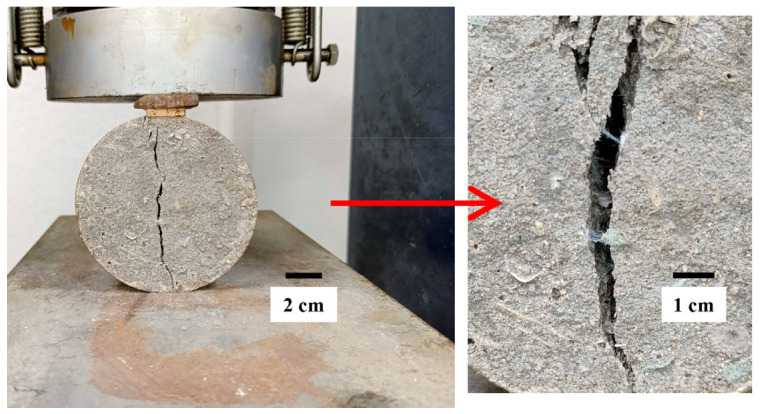
Micro-bridge effect in mortar FM3-0.2.

The split tensile strength test results showed that for FM contents of up to 0.2%, the highest strength was achieved in each group of mortars (Figure 14). This occurred because, at this percentage, the strip arrangement in the mortar is sufficient to minimize entanglement, and the pore volume does not significantly affect tensile strength. Without excessive entanglement, the FM strips work like small bridges or micro-anchors between tension zones within the mortar (Figure 15), better distributing loads and intercepting microcracks [18,103,104,105]. Researchers like Koniorczyk et al. [9] reported that the effective dispersion of reinforcing fibers in concrete samples is essential for improving strength.

Comparing the Control mixture with Control + OSP, it is observed that the presence of 5% OSP generated an increase of 6.1% in resistance. Regarding the mixtures with strips, those of the FM1 group showed the highest resistance, with FM contents of 0.1% and 0.2%. These results indicate that for the shortest strip length (3 × 6 mm), a balance is achieved between the load distribution of the strips and the porosity generated by their inclusion.

For contents greater than 0.2% FM content, the resistance gradually decreased. This behavior became more evident as the strip length increased. For example, the FM3-0.8 mixture reduced its resistance by 40.8% compared to the Control + OSP sample. This decrease in resistance occurs due to the presence of greater porosity, poor distribution of the strips, and greater interlacing between them [23,106].

The *t*-test showed that for split tensile strength, there was no significant difference between the Control mortar and the mortar with OSP (*p* = 0.064, d = 1.178). The ANOVA analysis showed that both content (*p* < 0.001, η^2^ = 0.741) and fiber length (*p* = 0.002, η^2^ = 0.100) had a significant effect on the values of this strength. Again, the combination of both factors was not significant (*p* = 0.967, η^2^ = 0.008). The Tukey test showed that the contents of 0.1% and 0.2% were similar (*p* > 0.05). But when compared with contents of 0.5% and 0.8%, clear differences were observed (*p* < 0.05). For length, the results showed that mortars with FM1 did not present a significant difference with FM2 (*p* = 0.174) but did have a significant difference with FM3 mortars (*p* = 0.002). These results showed that split tensile strength is affected mainly by fiber content and, to a lesser extent, by fiber length.

### 3.7. Shear Bond Strength (SBS)

**Figure 16:**
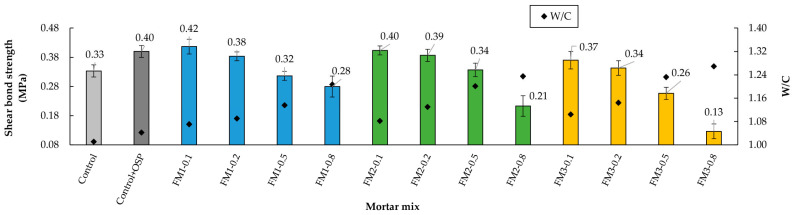
Shear bond strength of mortars with the inclusion of FM.

**Figure 17:**
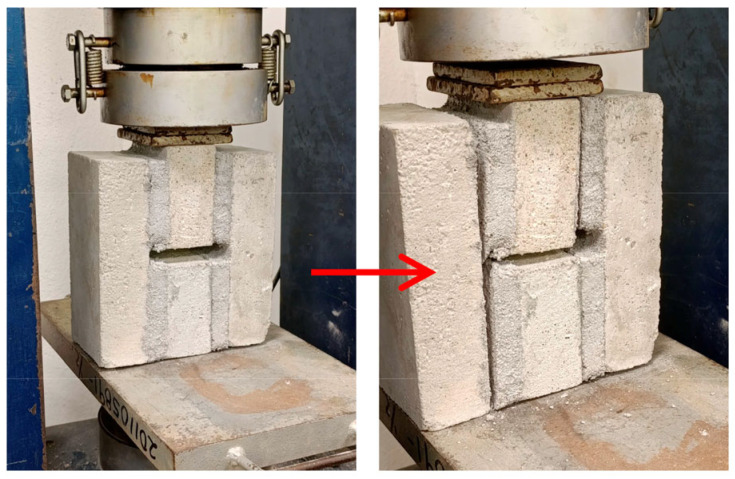
Adhesive failure during all the SBS tests in mortars (FM2-0.2).

Figure 16 shows that, in general, increasing the number of FM strips reduced the SBS. This trend intensified as strip length increased. Analyzing mortars with the same FM content, the type with the highest SBS was FM1, while FM3 mortars showed the lowest SBS. For FM1 and FM2 mortars, SBS remained relatively close to the Control + OSP mortar’s value, up to a strip content of 0.2%. Comparing the mixtures without FM reinforcement, it is observed that the Control + OSP sample increased the SBS by 21%. This is due to the filling effect of the OSP, which reduces the pores and increases the contact area at the mortar-brick interface. In all mixtures tested in this investigation, an adhesive-type failure was observed. Figure 17 shows this failure in an M2-0.2 sample.

The progressive reduction in SBS with increasing FM content was mainly due to two factors. The first is the increase in air content, since the bubbles that are trapped in the mixture reduce the effective contact area between the mortar paste and the brick, thus limiting adhesion. Additionally, the decrease in density (Section 3.2) caused by the increase in air content causes a decrease in solid material per unit volume, further reducing the effective contact area. The second factor is linked to the very nature of FM: its hydrophobic surface leads to poor bonding with the mortar paste, creating areas of weakness [97]. Furthermore, when the strips themselves within the cement paste come into direct contact with the brick, they reduce the adhesion area. Ajam et al. [23] demonstrated that when FM strips are cut with their three layers joined together, the mortar paste cannot penetrate between them, which generates porosity and areas of very low adhesion.

The *t*-test showed that regarding SBS (n = 5 specimens per mixture, as described in the Testing Program), there was a significant difference between the Control mortar and the mortar with OSP (*p* = 0.016, d = −3.266). This indicates that adding OSP improved mortar adhesion, probably due to better particle arrangement at the adhesion interface. The ANOVA analysis showed that both content (*p* < 0.001, η^2^ = 0.742) and fiber length (*p* < 0.001, η^2^ = 0.145) had a significant effect on this property. The interaction between both factors was also significant (*p* = 0.007, η^2^ = 0.056). This indicates that the influence of fiber content on SBS changes depending on strip length, and strip length also modifies the effect of content. The Tukey test showed that the contents of 0.1% and 0.2% were similar (*p* > 0.05), while when compared with 0.5% and 0.8%, clear differences were found (*p* < 0.001). Regarding length, mortars with FM1 were not different from those with FM2 (*p* = 0.36) but were different from those with FM3 (*p* < 0.001). These results allow the conclusion that in SBS development, fiber content and length are determining factors, and that in this case, both factors interact with each other.

## 4. Conclusions

The experimental results obtained in this study provide clear insights into the behavior of mortars modified with disposable face mask (FM) strips and calcined oyster shell powder (OSP). These conclusions address both the technical performance and the potential for waste valorization in eco-friendly construction:

- The addition of 5% OSP increased compressive strength by approximately 10% and improved shear bond strength, demonstrating its potential as a reactive filler and its contribution to matrix densification.
- Increasing the FM strip content progressively raised the air content of the mixtures, which negatively affected most of the properties evaluated.
- Increasing strip length produced three main effects: (1) higher air content due to the hydrophobic nature of the strip surface; (2) greater tendency for entanglement, generating additional porosity and creating weak zones within the matrix; and (3) greater interconnection between pores, facilitating water migration through the mortar.
- Under controlled conditions, FM strips acted as micro-bridges or micro-anchors between tensile zones, improving load distribution.
- The optimal content for balanced performance across the three series of mixes was 0.2%, a percentage that ensures proper strip dispersion, minimizes entanglement, and prevents the generated porosity from significantly affecting the evaluated properties.
- With 0.2% content and dimensions of up to 3 × 6 mm (width/length ratio = 0.5), splitting tensile strengths close to the Control + OSP mortar were achieved, indicating that this length favors tensile load transfer within the matrix.

This study focused on technically validating the recycling process of FMs as reinforcing elements and oyster shells for their potential use as fillers in mortars. In future stages, this process can be optimized, including the calcination stage of the shell, in order to reduce the associated CO_2_ emissions.

Recommendations for future research

- Assess long-term durability under marine and urban exposure conditions.
- Investigate air-reducing admixtures and optimized mixing procedures to improve density and mechanical performance.
- Explore surface treatments or alternative geometries for FM strips to enhance adhesion and reduce entanglement.
- Incorporate microstructural characterization techniques to better understand how fillers and fibers interact with cement paste.
- Evaluate and optimize the calcination stage to reduce CO_2_ emissions associated with the process.

## Figures and Tables

**Table 1:** Proportions of the mixes used.

Mortar Mix	Face Mask Type	FM (%)	NA (g)	CEM (g)	Filler (g)	Water (g)	Consistency Index—Flow Diameter (mm)	W/C
Control	—	0	1573	353	0	357	176	1.011
Control + OSP	—	0	1573	353	17.65	368	177	1.042
FM1-0.1	FM1 (3 × 6 mm)	0.10	1573	353	17.65	378	175	1.071
FM1-0.2		0.20	1573	353	17.65	385	171	1.091
FM1-0.5		0.50	1573	353	17.65	401	178	1.136
FM1-0.8		0.80	1573	353	17.65	426	170	1.207
FM2-0.1	FM2 (3 × 15 mm)	0.10	1573	353	17.65	382	170	1.082
FM2-0.2		0.20	1573	353	17.65	399	173	1.130
FM2-0.5		0.50	1573	353	17.65	424	175	1.201
FM2-0.8		0.80	1573	353	17.65	436	176	1.235
FM3-0.1	FM3 (3 × 28 mm)	0.10	1573	353	17.65	390	175	1.105
FM3-0.2		0.20	1573	353	17.65	404	171	1.144
FM3-0.5		0.50	1573	353	17.65	435	178	1.232
FM3-0.8		0.80	1573	353	17.65	448	170	1.269

Notes: NA: natural aggregate; CEM: cement; OSP: oyster shell powder; FM: face mask strips; W/C: water-to-cement ratio. All mixtures except the Control contained 5% OSP by dry weight of cement.

## Data Availability

The original contributions presented in this study are included in the article. Further inquiries can be directed to the corresponding author.
